# A single and rapid calcium wave at egg activation in *Drosophila*

**DOI:** 10.1242/bio.201411296

**Published:** 2015-03-06

**Authors:** Anna H. York-Andersen, Richard M. Parton, Catherine J. Bi, Claire L. Bromley, Ilan Davis, Timothy T. Weil

**Affiliations:** 1 Department of Zoology, University of Cambridge, Downing Street, Cambridge CB2 3EJ, UK; 2 Department of Biochemistry, University of Oxford, South Parks Road, Oxford OX1 3QU, UK; 3 MRC Centre for Developmental Neurobiology, King's College London, London SE1 1UL, UK

**Keywords:** Egg activation, Calcium imaging, *Drosophila*, Calcium transient

## Abstract

Activation is an essential process that accompanies fertilisation in all animals and heralds major cellular changes, most notably, resumption of the cell cycle. While activation involves wave-like oscillations in intracellular Ca^2+^ concentration in mammals, ascidians and polychaete worms and a single Ca^2+^ peak in fish and frogs, in insects, such as *Drosophila*, to date, it has not been shown what changes in intracellular Ca^2+^ levels occur. Here, we utilise ratiometric imaging of Ca^2+^ indicator dyes and genetically encoded Ca^2+^ indicator proteins to identify and characterise a single, rapid, transient wave of Ca^2+^ in the *Drosophila* egg at activation. Using genetic tools, physical manipulation and pharmacological treatments we demonstrate that the propagation of the Ca^2+^ wave requires an intact actin cytoskeleton and an increase in intracellular Ca^2+^ can be uncoupled from egg swelling, but not from progression of the cell cycle. We further show that mechanical pressure alone is not sufficient to initiate a Ca^2+^ wave. We also find that processing bodies, sites of mRNA decay and translational regulation, become dispersed following the Ca^2+^ transient. Based on this data we propose the following model for egg activation in *Drosophila*: exposure to lateral oviduct fluid initiates an increase in intracellular Ca^2+^ at the egg posterior via osmotic swelling, possibly through mechano-sensitive Ca^2+^ channels; a single Ca^2+^ wave then propagates in an actin dependent manner; this Ca^2+^ wave co-ordinates key developmental events including resumption of the cell cycle and initiation of translation of mRNAs such as *bicoid*.

## Introduction

The eggs of different species of animals are arrested at distinct stages of meiosis and egg activation is required in all species to transform the egg to a cell capable of initiating embryogenesis following fertilisation. Conserved changes in the egg at activation include: adjusting the composition of the outer membranes and egg shell; cortical granule exocytosis, also known as cortical reaction; the resumption of meiosis via inactivation of MPF and activation of APC, mRNA translation activation or degradation, as well as cytoskeletal rearrangements (Horner and Wolfner, 2008; Houston, 2012; Krauchunas and Wolfner, 2013). In most animals studied to date it has been shown that changes in intracellular Ca^2+^ concentrations play a major role in setting these events in motion (Krauchunas and Wolfner, 2013). Ca^2+^ is a well-known second messenger involved in many signal transduction cascades in cells and tissues co-ordinating muscle contraction, transport processes, cell division and growth and enzyme activities (Berridge, 2005; Clapham, 2007). Intracellular Ca^2+^ concentrations are kept highly regulated at approximately 100 nM as prolonged exposure to high levels of Ca^2+^ is toxic to the cell. As a result, there is typically a large difference in the Ca^2+^ concentration between the internal and external cellular environments and signalling events are transient and/or restricted spatially. This is maintained by several ATP-dependent pumps and storage of Ca^2+^ in intracellular organelles, such as the endoplasmic reticulum (ER) or mitochondria (Berridge, 2005; Clapham, 2007).

The specific spatiotemporal dynamics of the transient increases in cytoplasmic Ca^2+^ have been characterised across many species during egg activation and reveal important differences between species (Stricker, 1999). In *Drosophila*, despite genetic evidence that Ca^2+^ signalling is required at egg activation (Horner et al., 2006), to date, a Ca^2+^ transient has not been observed. Unlike many animals, *Drosophila* do not require fertilisation to trigger activation (Doane, 1960). *In vivo* experiments in *Drosophila* have shown that activation occurs during the transition from the ovary to the oviduct prior to fertilisation in the uterus (Heifetz et al., 2001). Egg activation relies entirely upon maternal factors that, without the addition of the sperm, can trigger the *Drosophila* egg to complete meiosis (Doane, 1960), modify the vitelline membrane (Heifetz et al., 2001) and initiate translation or degradation of some mRNAs (Macdonald and Struhl, 1986; Tadros and Lipshitz, 2005).

With egg activation taking place independently of fertilisation in *Drosophila*, both mechanical stimulation during the passage of the egg through the female reproductive tract and osmotic pressure from the fluid composition in the oviduct have been proposed as a trigger of activation (Went and Krause, 1973; Heifetz et al., 2001; Horner and Wolfner, 2008). *Ex vivo* experiments on *Drosophila* eggs show that physical pulling on the dorsal appendages can cause the resumption of meiosis (Endow and Komma, 1997), placing them into a hypotonic buffer causes them to swell and activate (Mahowald et al., 1983; Page and Orr-Weaver, 1997) and more recently, osmotic and hydrostatic pressure can activate the egg (Horner and Wolfner, 2008). Before activation, mature eggs appear shrivelled and dehydrated, whereas following activation in the female or *ex vivo* with activation buffer, eggs are turgid and enlarged (Mahowald et al., 1983). Furthermore, evidence shows that the oviduct matrix is more hydrated in virgin females that have undergone ovulation, compared to that in females in the process of ovulating (Mahowald et al., 1983), raising the possibility that fluid is transferred to the egg from the oviduct matrix during ovulation.

While the mechanism of triggering egg activation is unclear, the importance of Ca^2+^ signalling is well established. Mutations in the *Drosophila* calcipressin *sarah* (*sra*), part of a pathway with the Ca^2+^-dependent phosphatase calcineurin, result in female sterility, cell cycle arrest and defects in *bicoid* (*bcd*) mRNA translation (Horner et al., 2006; Takeo et al., 2006). This pathway represents a conserved pathway between *Drosophila*, sea urchins and some vertebrates (Ducibella and Fissore, 2008; Horner and Wolfner, 2008).

In order to establish definitively, whether changes occur in intracellular Ca^2+^ in *Drosophila* eggs, and subsequently to test how these changes are co-ordinated, we utilised micro-injected Ca^2+^ detecting dye and a genetically encoded Ca^2+^ indicator. We identified a single, rapid Ca^2+^ wave passing through the mature oocyte. Genetic, pharmacological and physical manipulation reveals that the Ca^2+^ wave is not initiated by pressure on the egg alone but is dependent on both a functional actin cytoskeleton and the calcineurin signalling pathway. Our data supports a model where the fluid in the lateral oviduct, which rehydrates the egg, triggers Ca^2+^ wave initiation at the posterior through mechano-sensitive channels. Finally, we show that processing (P) bodies are dispersed following the Ca^2+^ wave and propose that this is required to activate the translation of stored mRNAs in the egg as a response to activation.

## Materials and Methods

### Fly strains

Stocks were raised on standard cornmeal–agar medium at 21 or 25°C: wild-type, OregonR; UASt myristoylated *(myr)-GCaMP5* (Melom and Littleton, 2013); GAL4::VP16-nosUTR (Van Doren et al., 1998); *sarah^A108^*; *sarah^A426^* (Horner et al., 2006); Me31B::GFP (Buszczak et al., 2007). For all experiments, mated females were fattened on yeast for 24–48 hours at 25°C.

### Preparation of egg chambers for imaging

For live imaging, oocytes were prepared as described previously (Weil et al., 2012). Ovaries from well-fed females were dissected and individual egg chambers separated from ovarioles using a dissecting probe in series95 halocarbon oil (KMZ chemicals) on 22 × 40 cover slips, #1.5 (Menzel-Gläser). Buffer was then added by glass pipette on top of the sample. Precise start times are not always clear due to variability in oil displacement sample to sample. Furthermore, the propagation of the applied medium was not always consistent. As far as possible these variables was controlled for by selecting the starting time point based on bright-field images.

### Embryo collection

As described previously (Parton et al., 2010), embryos were collected at 25°C between 0–4 hours on yeasted apple juice agar plates then dechorionated for 2 minutes in 50% bleach. For imaging, embryos were adhered with heptane glue to a #1.5 coverslip then covered with series700 halocarbon oil (KMZ chemicals).

### Microinjection

Preparation for microinjection was carried out as described previously (Weil et al., 2012). Stage 14 egg chambers were prepared as above. A 2×10 mm piece of glass was placed on the coverslip to unblock the needle if required. A Femtotips II microinjection needle (Eppendorf) and a gas pressure injection system (Tritech Research) were used to inject the egg chambers. Dye aqueous stock solutions were made at 1 mM Calcium Green-1 Dextran, Mr 10,000 (Invitrogen) and 1 mM Texas Red Dextran, Mr 10,000 (Invitrogen). For ratiometric calcium imaging, dye stocks were mixed 1:1 then briefly centrifuged at high speed prior to loading into the injection needle and injection. After injection, 45 minutes was left for recovery and dye diffusion before addition of activation buffer. Calcium Green-1 K_d_ for Ca^2+^ = 190 nM. For artificial elevation of cytoplasmic Ca^2+^ levels, 20–100 pl of 10 mM CaCl_2_ was injected.

### Solutions, pharmacological treatments and *ex vivo* activation

To activate the oocytes *ex vivo*, a standard hypotonic activation buffer, first developed by Mahowald and colleagues (Mahowald et al., 1983) and also described by Page and Orr-Weaver (Page and Orr-Weaver, 1997), was used, composed of: 3.3 mM NaH_2_PO_4_, 16.6 mM KH_2_PO_4_, 10 mM NaCl, 50 mM KCL, 5% polyethylene glycol 8000, 2 mM CaCl_2_, brought to pH 6.4 with a 1:5 ratio of NaOH:KOH. Other solutions used include Schneider's Insect medium (Sigma) for control experiments, 10 mM CaCl_2_ and activation buffer with 10 µg/ml final concentration cytochalasin-D (Sigma).

### Imaging

Ratiometric imaging was performed with a wide-field DeltaVision CORE microscope (GE-Applied Precision, Olympus Ix70 microscope, 20× 0.75NA dry lens, Roper Scientific CascadeII ECCD camera). Ca^2+^ measurements were performed using two fluorescent dyes- Calcium Green-1 Dextran (Ca^2+^ sensitive) and Texas Red Dextran (Ca^2+^ insensitive volume marker). Ratio image calculation was performed in Fiji (Image J) and displayed in pseudocolour. All other images were acquired with an inverted Leica SP5 Confocal microscope under a 20× 0.7NA immersion objective. Experiments requiring mechanical pressure utilised a mounted microinjection apparatus (Olympus). Myr-GCaMP5 was driven by the Gal4/UAS system. GCaMP5 K_d_ for Ca^2+^ = 660 nM (Melom and Littleton, 2013). Fiji (Image J) was used to manipulate the images for the figures.

## Results

### A single transient Ca^2+^ wave accompanies egg activation in *Drosophila*

**Fig. 1.**
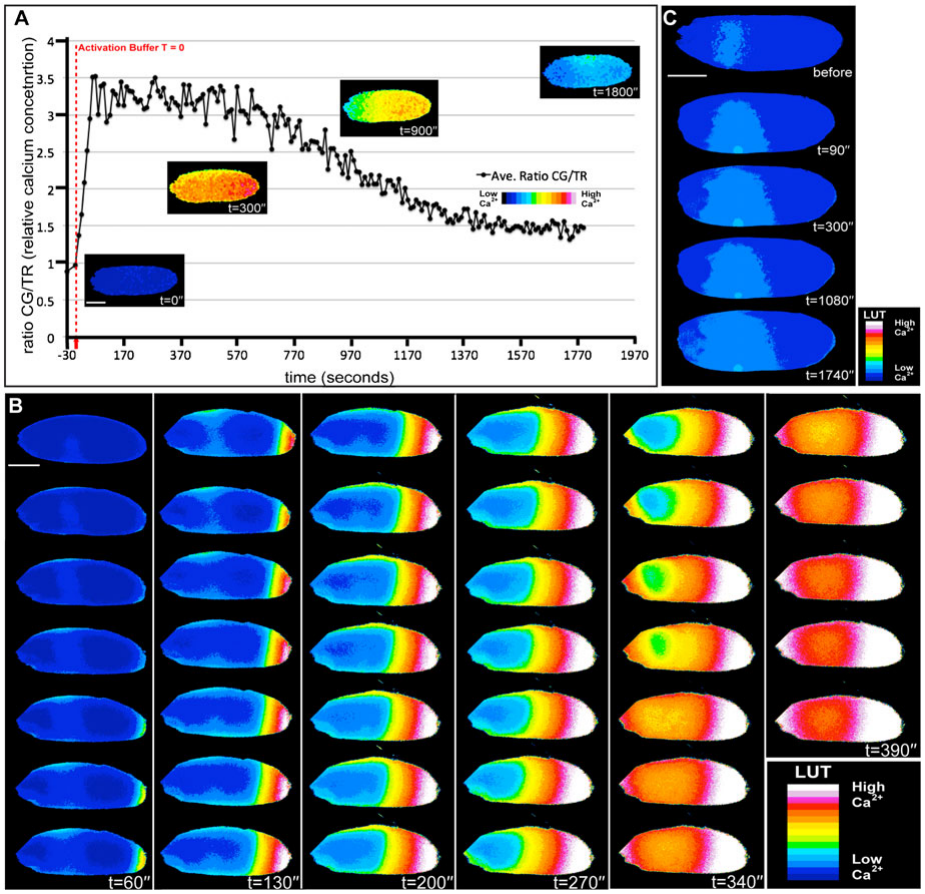
A rapidly propagating Ca^2+^ wave at egg activation. (A–C) Mature oocytes microinjected with Calcium Green-1 and Texas Red Dextrans and left for dyes to diffuse for 45 minutes. (A) Addition of activation buffer results in a rapid increase in intracellular Ca^2+^ within the first 90 seconds followed by a slower recovery. Graph displays a single representative egg chamber imaged in a single cortical plane in order to calculate relative increase in calcium in the entire egg. Plotted values are the average for the entire egg chamber. (B) Time series showing propagation of the Ca^2+^ transient at high temporal resolution, cross-section images displayed every 10 seconds. (C) The addition of Schneider's Insect medium (t = 0′) shows no propagating Ca^2+^ response. Scale bars A–C = 100 µm. Time in minutes (′) and seconds (″).

Although mutations in the Ca^2+^ signalling pathway have been shown to disrupt egg activation (Horner et al., 2006), to date, Ca^2+^ signalling events have not been recorded during *Drosophila* egg activation. To investigate this we monitored the change in intracellular Ca^2+^ by ratiometric imaging on *ex vivo* isolated mature oocytes (stage 14). Prior to addition of activation buffer, both Calcium Green-1 Dextran (10 kDa) and Texas Red Dextran (10 kDa) were micro-injected in equal concentrations and allowed to evenly diffuse through the cytoplasm (45 minutes) to provide a baseline measure of intracellular Ca^2+^ concentration. Upon activation, we observed a consistent, rapidly propagating increase in intracellular Ca^2+^, followed by a slower “recovery phase”, decrease (Fig. 1A,B, n = 8). These events were concomitant with the physical “swelling” response normally associated with activation (data not shown). To confirm that the change in Ca^2+^ was due to activation rather than simply mechanical perturbation, Schneider's Insect medium was applied to the post-injected egg chambers (Fig. 1C, n = 8). In these cases there was no Ca^2+^ wave or swelling, however, there was a small local Ca^2+^ elevation associated with the injection site, which did not propagate. We verified that the ratio imaging method was responding to Ca^2+^ by artificially elevating the Ca^2+^ concentration locally through microinjection of CaCl_2_ (data not shown).

### The Ca^2+^ transient rapidly propagates as a wave from the cortical posterior

**Fig. 2.**
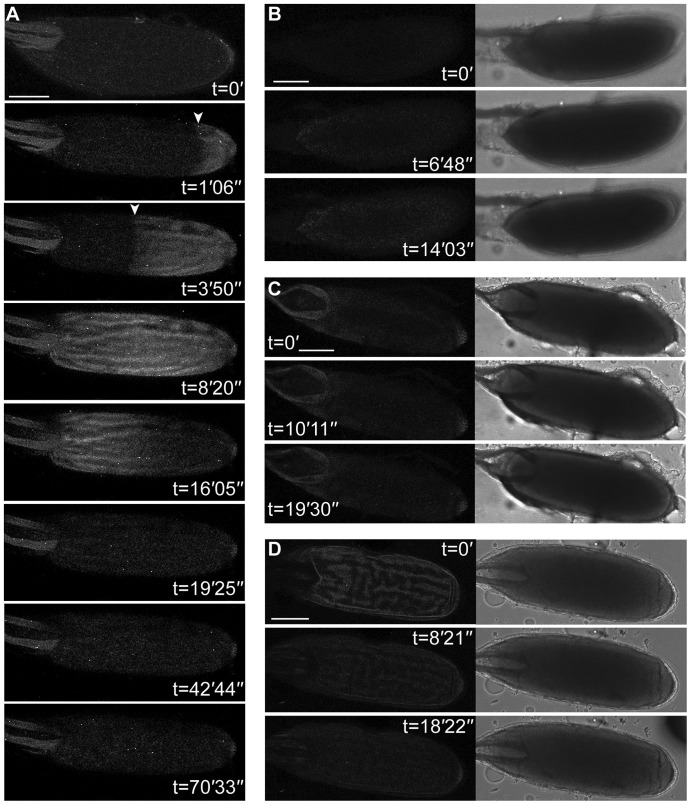
A rapidly propagating Ca^2+^ response at egg activation detected by transgenic indicator. (A) Time series of a mature oocyte expressing UAS-*myrGCaMP5* following the addition of activation buffer, (t = 0′) shows an expected baseline of fluorescence (note: dorsal appendages to the left show typical autofluorescence). After the addition of activation buffer, the mature oocyte swells and a wave of increases Ca^2+^concentration initiates from the posterior pole (t = 1′06″, arrowhead). The posterior wave propagates across the mature oocyte (t = 3′50″, arrowhead). Following a brief period when the whole cell has an increase in Ca^2+^ (t = 8′20″), a slower recovery commences (t = 16′05″) and leaves the cell a similar Ca^2+^ concentration as prior to addition of activation buffer (t = 19′25″) and no oscillations are detected. (B) Time series of a mature oocyte not expressing UAS-*myrGCamp-5* (without the tub-GAL4VP16 driver) or (C) wild-type following the addition of activation buffer. Corresponding bright-field images show the mature oocytes swelling and fluorescent images show no change in fluorescence, as expected (n = 7). (D) Mature oocytes expressing tub-GAL4VP16 and UAS-*myrGCamp-5* following the addition of Schneider's Insect media does not show the mature oocyte swelling and an increase in Ca^2+^ is not detected (n = 7). Scale bars A–D = 100 µm. Max projection A = 41 µm, B = 40.3 µm, C = 43.7 µm, D = 33.2 µm.

While injected dyes allowed us to initially observe and make preliminary characterisation of the calcium response we found it difficult to thoroughly analyse speed and magnitude. To overcome this and in order to further characterise the Ca^2+^ transient during activation by standard hypotonic activation buffer, we used the genetically encoded Ca^2+^ indicator Ca^2+^-sensitive GFP (GCaMP) (Nakai et al., 2001) under the control of a germ line specific GAL4 driver. We found that using the myristoylated variant of the indicator (Melom and Littleton, 2013), targeting it to the inner leaflet of the plasma membrane, resulted in a dramatic increase in the signal-to-noise ratio between the GFP and auto-fluorescent yolk granules in the cytoplasm of the egg. This approach showed a similar transient Ca^2+^ increase to that detected with injected dyes (Fig. 1B, Fig. 2A; supplementary material Movies 1–3). Using UAS-*myrGCaMP5*, we could clearly see that the Ca^2+^ transient propagated as a wave (Fig. 2A, arrowheads) and subsequently returned to basal levels (Fig 2A). Initiation of the wave following the addition of activation buffer was observed on average at 1′15″ ± 17.5″ (n = 26, SEM) and the completion of the wave resulting in the whole oocyte showed an increase in intracellular calcium after 3′ 35″ ± 41.1″ (n = 16, SEM). Recovery begins 4′06″ ± 50.6″ (n = 10, SEM) later and took 5′33″ ± 50″ (n = 10, SEM) to complete. The majority of waves initiated from the posterior pole (69%) versus the anterior (18%) or lateral cortex (13%) (n = 29). Even with prolonged observations, we did not detect any further transients or oscillating waves after ex-vivo activation (Fig. 2A, t = 70′) or in the early embryo (0–2 hours) (data not shown).

Expression of the UAS-*myrGCaMP5* Ca^2+^ reporter did not interfere with the expected morphological changes in the oocyte associated with activation, including swelling, rounding at the poles and increased rigidity (supplementary material Movie 1), which were similar to control experiments where the reporter was not expressed (Fig. 2B, data not shown). In these control situations, no change in fluorescence was detected consistent with the lack of indicator (Fig. 2C, contrast adjusted to highlight autofluorescence levels). Furthermore, we did not observe any morphological or Ca^2+^ changes, when standard insect media was added to oocytes expressing driven UAS-myrGCaMP5 in place of activation buffer (Fig. 2D).

### External Ca^2+^ alone does not trigger a propagating Ca^2+^ wave

**Fig. 3.**
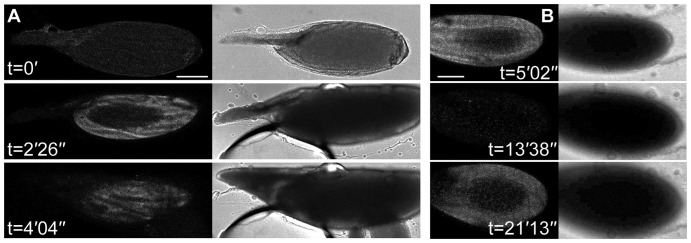
Osmotically induced swelling of the mature oocyte results in an increase in Ca^2+^ and lysis. (A,B) Time series of a mature oocyte expressing UAS-*myrGCaMP5* following the addition of 10 mM CaCl_2_ (A) or water (B). (A) Addition of 10 mM CaCl_2_ results in a rapid cortical increase in intracellular Ca^2+^, accelerated swelling and lysis (n = 8). (B) Addition of water causes a rapid increase in intracellular Ca^2+^ around the circumference of the cell (n = 10). The cell recovers to pre-activation levels before showing second increase in intracellular Ca^2+^ before lysis. Corresponding bright-field images show a continued swelling of the mature oocyte until lysis. Scale bars A,B = 100 µm. Max projection A = 27.7 µm, B = 41 µm.

In order to better understand the basis of the Ca^2+^ wave during activation by standard hypotonic activation buffer, we tested if the application of solutions with different properties had an effect on intracellular Ca^2+^ in the egg. We already showed that Schneider's Insect medium (5.4 mM CaCl_2_, 2.7× more CaCl_2_ than in activation buffer) did not trigger a Ca^2+^ transient or activation. However, addition of 10 mM CaCl_2_, which is five times more concentrated than the CaCl_2_ in activation buffer, caused a rapid increase in intracellular Ca^2+^ from all regions of the cortex, accelerated swelling and lysis within minutes (Fig. 3A). Next, we demonstrated that altering the membrane potential, through the application of a high potassium medium (Ataman et al., 2008), caused no swelling or intracellular Ca^2+^ change (data not shown). Finally, we showed that hypo-osmotic shock by addition of dH_2_O promotes accelerated swelling as well as an increase in Ca^2+^ in the egg (Fig. 3B). Interestingly, with the addition of water we often observe a recovery of intracellular Ca^2+^ to normal levels and occasionally a second increase in Ca^2+^ (Fig. 3B). Together, this data suggests that mechanical pressure from hypo-osmotically induced swelling is a likely mechanism for initiation of the Ca^2+^ wave, possibly initially triggered by entry of external Ca^2+^. The data also shows that while the egg chamber is clearly capable of multiple transients, activation involves only a single propagating wave.

### Mechanical stimulation alone does not support the propagating Ca^2+^ wave

**Fig. 4.**
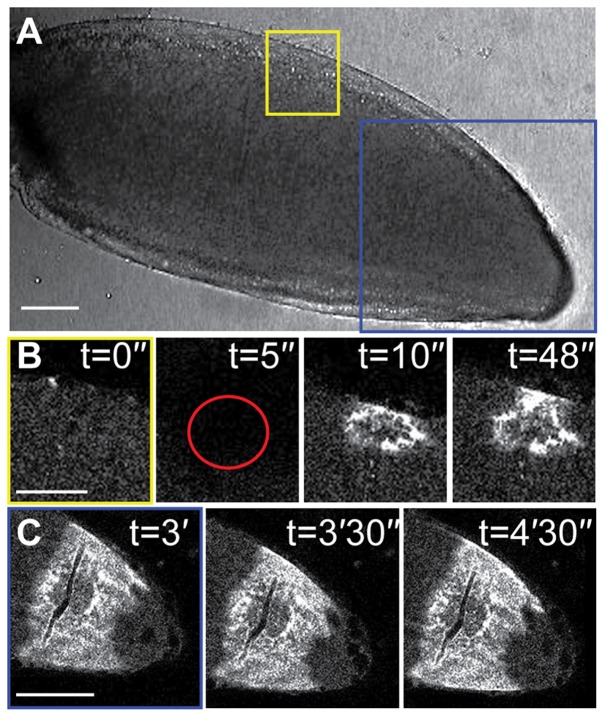
Local pressure causes an increase in Ca^2+^ but not a propagation of the wave. (A–C) Mature oocyte expressing UAS-*myrGCaMP5* having local pressure applied (n = 8). (A) Corresponding bright-field image of the mature oocyte with boxes highlighting the regions where pressure was applied. (B) Direct pressure from the flat side of a glass rod (red circle, t = 5″) results in a local increase in intracellular Ca^2+^ concentration but does not cause the cell to swell as is normally associated with egg activation. (C) Pressure applied (t = 0′) locally does not propagate across the cell. Scale bars A,B = 50 µm, C = 100 µm. A–C single frame.

**Fig. 5.**
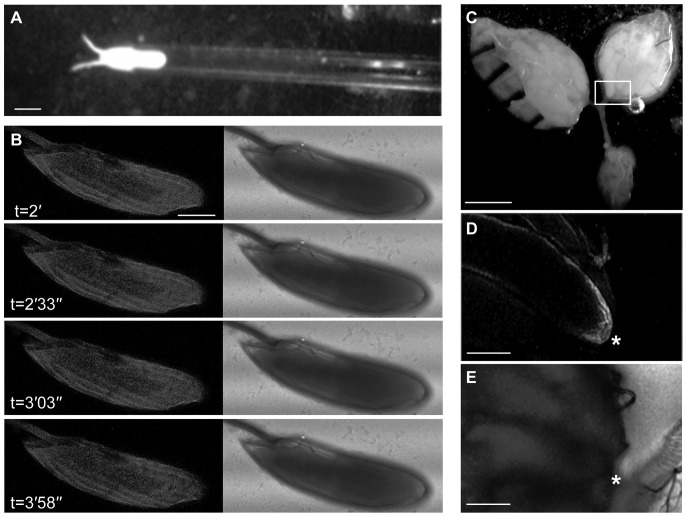
Suction alone is not sufficient to cause a Ca^2+^ wave. (A,B) Time series of a mature oocyte expressing UAS-*myrGCamp-5* dissected into Schneider's Insect medium with suction applied to the posterior half of the mature oocyte with a 135 µm diameter plastic tube for 2 minutes (A). Subsequent time series show no morphological or intracellular Ca^2+^ changes (B) (n = 7). (C) Whole female reproductive machinery dissection, white box highlights the region of interest for experiment in panels D and E. (D,E) Higher magnification of whole female reproductive machinery expressing UAS-*myrGCamp-5* cultured in Schneider's Insect medium imaged with fluorescence (D) and corresponding bright-field image (E). A mature oocyte preparing to enter or slightly entered the lateral oviduct (white star) shows an increase in intracellular Ca^2+^ at the posterior pole (n = 3 due to technical challenges in imaging live at this magnification). Scale bars A,B,D,E = 100 µm, C = 1000 µm. Max projection B = 40 µm.

It has long been postulated that both mechanical stimulation and external cues contribute to *Drosophila* egg activation during deposition (Heifetz et al., 2001; Horner and Wolfner, 2008; Krauchunas and Wolfner, 2013). Various methods have previously been used to study this question, for example, physical pulling on the dorsal appendages of mature oocytes has been shown to cause the resumption of meiosis (Endow and Komma, 1997) and placing mature oocytes into a hypotonic buffer causes them to swell and activate (Mahowald et al., 1983; Page and Orr-Weaver, 1997). To test whether mechanical pressure is able to prompt and propagate a wave of Ca^2+^, we applied pressure with a glass rod to the outside of an oocyte mounted in halocarbon oil (Fig. 4A,B). Despite a local increase in Ca^2+^ where pressure was applied, no propagating wave or physical hallmarks of activation were observed (Fig. 4C). To better represent the physiological process of the passage of an egg chamber through the oviduct, we applied pressure on the *ex vivo* mature oocyte with plastic tubes, normally used for cell isolation prior to IVF treatments (Fig. 5A,B). We show that cylindrical pressure for 1–3 minutes on the posterior half of the mature oocyte did not result in a Ca^2+^ or physical change (Fig. 5A,B). These results further support the model that the external environment, likely the fluid in the oviduct (Heifetz et al., 2001) is required for initiation of a Ca^2+^ wave.

### Ca^2+^ transients occur in oocytes passing through the *ex vivo* oviduct

Amongst the earliest changes associated with activation occur upon entrance of the oocyte into the lateral oviduct, for example an increase in the cross-linking of the vitelline membrane (Heifetz et al., 2001). In order to test whether Ca^2+^ signalling is associated with these early physiological events, we dissected the full reproductive machinery of the female and observed mature oocytes that were at the entrance of the lateral oviduct (Fig. 5C–E). Despite extensive imaging challenges, we were able to detect an increase in Ca^2+^ at the posterior of some oocytes as they entered the lateral oviduct (Fig. 5D). These results confirm the Ca^2+^ transient as part of the normal process of egg activation in *Drosophila*.

### Requirements for initiation and propagation of the Ca^2+^ transient

**Fig. 6.**
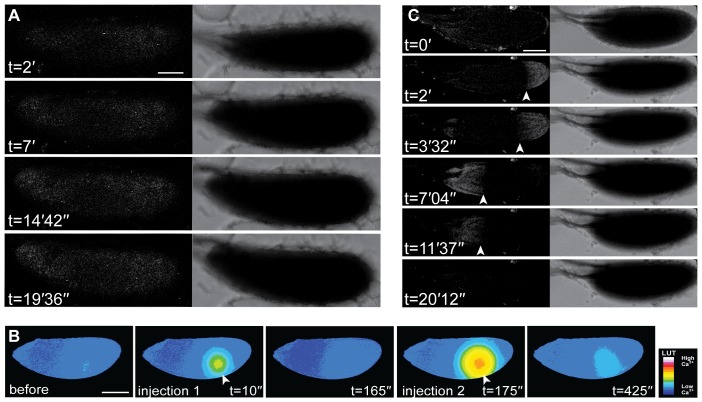
Ca^2+^ wave is compromised in *sra* mutant and by actin disruption. (A) *sarah* mutant mature oocyte expressing UAS-*myrGCamp-5*. Addition of activation buffer (t = 0′) does not show a change in intracellular Ca^2+^, while swelling and showing physical changes associated with egg activation (n = 13/14). (B) Mature oocyte microinjected with Calcium Green-1 and Texas Red Dextrans and left for dyes to diffuse for 45 minutes. A low volume injection of 10 mM CaCl_2_ (t = 10″, white arrowhead) shows a slight local increase in the Ca^2+^ concentration. Higher volume injection of 10 mM CaCl_2_ (t = 175″, white arrowhead) shows a clear local rise in the intracellular concentration of Ca^2+^ detected. Continued observation shows that the local increase in Ca^2+^ does not cause a wave to propagate in the mature oocyte. (C) Wild-type mature oocyte expressing UAS-*myrGCamp-5* cultured in activation buffer with 10 µg/ml cytochalasin-D (n = 21/24 show a complete loss or compromised wave). Intracellular Ca^2+^ increases from the posterior pole as in wild-type (t = 2′, white arrowhead). This posterior wave fails to move across the entire cell (t = 3′32″, white arrowhead), retracting to the posterior prematurely. A similarly compromised anterior wave fails to propagate fully (t = 7′04″, white arrowhead). Corresponding bright-field images show that swelling and separation of dorsal appendages occur as expected with egg activation. Scale bars A–C = 100 µm. Max projection A =  40 µm, C = 41.5 µm.

To explore the relationship between the Ca^2+^ wave and other aspects of egg activation, we tested mature oocytes mutant for the *Drosophila* calcipressin, *sra*, which has previously been shown to block release of the cell cycle (Horner et al., 2006; Takeo et al., 2006). We find that in a *sra* mutant background *ex vivo* activated mature oocytes swell normally but no Ca^2+^wave is detected in 13 of 14 samples (Fig. 6A). The fact that swelling and the initiation and propagation of the Ca^2+^ wave can be uncoupled in this way shows that swelling is not dependent upon the Ca^2+^wave and furthermore, swelling alone is not sufficient to initiate and drive the Ca^2+^ wave. Other factors must be required to couple the two events.

To test if a localised increase in Ca^2+^ alone could initiate and enable propagation of a wave, we injected CaCl_2_ directly. We show that both a low or high volume CaCl_2_ displays a proportional rise in the intracellular concentration of Ca^2+^ detected (Fig. 6B). We also show that despite this local increase, the cell recovers back to pre-injected levels and that the local increase in Ca^2+^ does not cause a wave to propagate in the mature oocyte.

Previous work has shown that the actin cytoskeleton is re-organised at activation (Weil et al., 2008). We tested if disruption of the actin cytoskeleton via pharmacological disruption could also block the Ca^2+^ wave. In the presence of activation buffer with cytochalasin-D, the wild-type egg swells, but the wave appears to stutter and retract prematurely, never encompassing the whole oocyte as observed in standard conditions (Fig. 6C; supplementary material Movie 4). These results demonstrate a role for the cytoskeleton in coupling swelling and propagation of the Ca^2+^ wave.

### The Ca^2+^ transient co-ordinates downstream events of egg activation

**Fig. 7.**
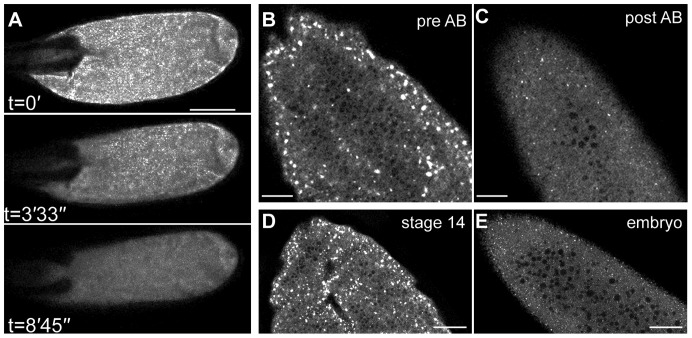
P bodies disperse at egg activation. (A–E) Mature oocytes and early embryo expressing Me31B::GFP labelling P bodies. (A) Time series of *ex vivo* mature oocyte following the addition of activation buffer (t = 0′) show Me31B foci dispersing. (B,C) Higher magnification 63× 1.4 NA lens before (B) and after (C) the addition of activation buffer. Large Me31B particles fall apart following the addition of activation buffer and swelling, consistent with observations of stage 14 oocyte (D) and early embryo (E). Scale bars A,B = 100 µm, C–E = 40 µm. Max projection A = 40 µm, B–E single frame.

Activation in *Drosophila* heralds a cascade of events, including translational initiation of several mRNA's: *bcd*, *nanos*, *hunchback*, *caudal*, *Toll*, *torso*, *smaug*, and *string* (Tadros and Lipshitz, 2005). Previous work has established the presence of cytoplasmic regions in the oocyte termed P bodies, where mRNA translation is not supported and which, therefore, act in repressing the translation of stored mRNA's such as *bcd* (Weil et al., 2012). Activation has previously been shown to dissipate P bodies at the anterior pole of the oocyte, releasing *bcd* mRNA for translation (Weil et al., 2012). To test the involvement of the Ca^2+^ transient in regulation of these P bodies and consequently in regulating translation regulation, we examined the common P body marker, Me31B, at activation (Fig. 7A–C). We show that Me31B particles present in the mature oocyte, disperse following *ex vivo* activation (n = 11). We observe a similar dispersed pattern of Me31B in the early embryo when compared to the large foci in stage 14 oocyte (Fig. 7D,E).

## Discussion

In this study we visualised a rapid wave of intracellular Ca^2+^ in the *Drosophila* oocyte at egg activation by two independent methods. We were able to demonstrate a requirement for the calcipressin encoded by the *sra* gene as well as actin for the initiation and propagation of the wave. We further show that mechanical pressure alone was insufficient to initiate and propagate a Ca^2+^ wave. Our data supports a model where at ovulation: (1) a stage 14 oocyte enters the lateral oviduct; (2) fluid in the oviduct initiates a change in intracellular Ca^2+^ at the posterior pole; (3) osmotic swelling and likely IP3 mediated release of intracellular Ca^2+^; (4) Ca^2+^ release by IP3 is in part controlled by a functional actin cytoskeleton; (5) a wave of Ca^2+^ propagates through the cell by Ca^2+^-induced Ca^2+^ release; (6) Ca^2+^ is reabsorbed into internal stores; (7) resumption of the cell cycle and translation of maternal mRNAs.

In most animals, Ca^2+^ release, in the form of a wave or oscillation, at egg activation is initiated by the sperm (Stricker, 1999). This results in cortical granule exocytosis to block polyspermy and the start of development though initiation of the cell's metabolism. Here we report a single rapid wave of Ca^2+^ in the *Drosophila* egg. This wave is unique with respect to other model systems insofar as it occurs prior to fertilisation but does appear to have similar properties to the waves observed in jellyfish and frogs. While gamete membrane fusion or binding of the gamete to receptors prior to fusion are common mechanisms to initiate activation, *Drosophila* must receive a different signal to set events into motion. One possibility is that a signal in the oviduct fluid bind to a receptor in the oocyte and triggers the tyrosine kinase, phospholipase C, phosphatidylinositol (4,5)-bisphosphate (PIP2), inositol 1,4,5-trisphosphate (IP3) cascade as in other systems (Berridge, 2005; Clapham, 2007), however this is not consistent with our *ex vivo* experiments.

While we cannot completely rule out a role for physical pressure or additional contribution from a specific factor for activation, our data supports a model where an increase in Ca^2+^ due to osmotic swelling enables initiation of activation. While direct injection of CaCl_2_ does not result in the propagating increase in calcium, we do observe that Schneider's media which enters the mature oocyte through the injection site can generate a sequential increase in intracellular Ca^2+^. This increase is not comparable in speed, time or distance covered to the normal wave we observe at activation. We observe initiation of the Ca^2+^ wave in *ex vivo* samples primarily from the posterior suggesting that there is something unique about this region of the cell. This could be due to a posterior enrichment of ER or Ca^2+^ channels. The former is unlikely as labelling for ER does not reveal any local enrichment (data not shown).

In sea urchin eggs, microinjection of purified IP3 is sufficient to cause cortical granule exocytosis (Busa et al., 1985; Whitaker and Irvine, 1984) and blocking IP3 prevents a Ca^2+^ release (Berridge, 2005; Clapham, 2007). In *Drosophila*, IP3 receptor germline clones fail to produce viable embryos and ovary extracts detect expression of the IP3 receptor (Acharya et al., 1997; Chintapalli et al., 2007; McQuilton et al., 2012). Our finding that disruption of the actin cytoskeleton results in a compromised Ca^2+^ wave hints at signalling through an actin-IP3 receptor interaction. We know from previous work that the F-actin cytoskeleton is rearranged at activation (Weil et al., 2008). Tissue culture, brain and pancreatic cells from mammals also show that actin physically interacts with the IP3 receptors (Fujimoto et al., 1995; Joseph and Samanta, 1993; Turvey et al., 2005). In starfish, disruption of actin resulted in a decrease in intracellular Ca^2+^ at egg activation (Kyozuka et al., 2008). Following what we hypothesise to be an actin dependent IP3 pathway release of Ca^2+^, it is likely that the Ca^2+^ would be reabsorbed into internal stores resulting in base levels of internal Ca^2+^ observed. An alternative is that the Ca^2+^ change could be mediated through the transient receptor potential and DEG/ENaC channel families which have been shown to be expressed in *Drosophila* adult ovary (Chintapalli et al., 2007; Horner and Wolfner, 2008; McQuilton et al., 2012). It is clear that further experimental work would be required to decipher which mechanisms are operating. Whatever the model, it is likely that the Ca^2+^ transient observed is important in regulating the subsequent events downstream of activation.

Now that we know that a calcium signalling event accompanies activation in *Drosophila*, this begs the question, which downstream events are triggered by the transient Ca^2+^ wave and how the events are activated. For example, the post-transcriptional regulation of maternal transcripts is critical to the subsequent development of the *Drosophila* egg post-activation (Tadros and Lipshitz, 2005; Tadros et al., 2007). The anterior determinant *bcd* mRNA shows Poly(A) elongation at activation in a *sra* dependent manner (Horner et al., 2006). Visualisation at activation has previously shown that *bcd* mRNA is released from anchoring in large aggregates at anterior pole and is no longer observed in P bodies (Weil et al., 2008; Weil et al., 2012). The loss of P bodies around the entire egg at activation could be a global mechanism for post-transcriptional regulation at egg activation. It remains unclear how Ca^2+^ mediates the observed changes in mRNA and P bodies. While Ca^2+^ signalling typically requires transaction proteins, such as calmodulin, another possibility is that Ca^2+^ mediates change through direct binding to its target. In this model, which is not restricted to *Drosophila* or egg activation, calcium bound to mRNA or P body components would thus alter their conformation, charge or association. Further examination of mRNA untranslated regions and protein dissociation in different calcium concentrations are required to test this hypothesis. *Drosophila*, with its advantages for live cell imaging and the availability of genetic tools will be an ideal system to approach these questions and determine whether similar mechanisms operate in other systems.

## Supplementary Material

Supplementary Material
