# Supplementary material for: A single and rapid calcium wave at egg activation in Drosophila
Source: Biol Open. 2015 Mar 6;4(4):553–60. doi: 10.1242/bio.201411296 (PMC4400597; doi:10.1242/bio.201411296)
Supplement: Supplementary Material [file supp_4_4_553__index.html]

A single and rapid calcium wave at egg activation in Drosophila — Supplementary Material 

# A single and rapid calcium wave at egg activation in *Drosophila*

## bio.201411296 Supplementary Material

**Files in this Data Supplement:**

- Supplementary Material - Anna H. York-Andersen et al. doi: 10.1242/bio.201411296
- Movie 1 - **A mature oocyte expressing UAS-*myrGCaMP5* following the addition of activation buffer.** Time series shows a wave of Ca2+ initiating from the posterior pole propagating over the whole cell. A slower recovery follows and no oscillations are detected for the rest of the observation (90 minutes). Z stack collected over 33 seconds Max projected 41 μm and played at 330 times normal speed (corresponding to Fig. 2A).
- Movie 2 - **A mature oocyte expressing UAS-*myrGCaMP5* following the addition of activation buffer.** A secondary anterior propagation of Ca2+ is detected and recovery initiated from the centre of the cell propagating outwards. Z stack collected over 12 seconds Max projected 32 μm and played at 120 times normal speed.
- Movie 3 - **A mature oocyte expressing UAS-*myrGCaMP5* following the addition of activation buffer.** Posterior initiation of the Ca2+ wave with secondary propagation from the anterior and lateral cortex. Recovery is initiated from both poles. Z stack collected over 20 seconds Max projected 38 μm and played at 200 times normal speed.
- Movie 4 - **A mature oocyte expressing UAS-*myrGCaMP5* cultured in activation buffer with 10 μg/ml cytochalasin-D.** Intracellular Ca2+ increases from the posterior pole as in wild-type but fails to propagate across the entire cell. A similarly compromised anterior wave also fails to propagate fully. Z stack collected over 30 seconds Max projected 41.5 μm and played at 300 times normal speed (corresponding to Fig. 6C).
