# Supplementary material for: A single and rapid calcium wave at egg activation in Drosophila
Source: Biol Open. 2015 Mar 6;4(4):553–60. doi: 10.1242/bio.201411296 (PMC4400597; doi:10.1242/bio.201411296)
Supplement: Supplementary Material [file supp_bio.201411296_bio.201411296-s1.pdf]

Biology Open (2015) 000, 1–8 doi:10.1242/bio.201411296

Supplementary Material
Anna H. York-Andersen et al. doi: 10.1242/bio.201411296

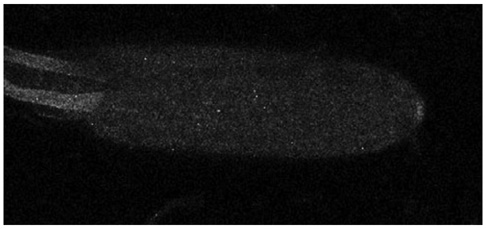

Movie 1. A mature oocyte expressing UAS-*myrGCaMP5* following the addition of activation buffer. Time series shows a wave of Ca^{2+} initiating from the posterior pole propagating over the whole cell. A slower recovery follows and no oscillations are detected for the rest of the observation (90 minutes). Z stack collected over 33 seconds [Max projected 41 µm] and played at 330 times normal speed (corresponding to Fig. 2A).

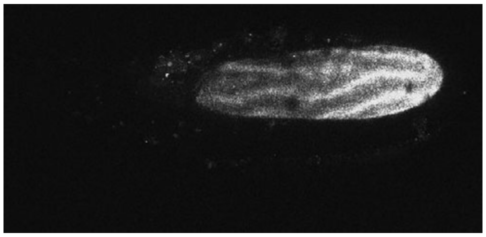

Movie 2. A mature oocyte expressing UAS-*myrGCaMP5* following the addition of activation buffer. A secondary anterior propagation of Ca^{2+} is detected and recovery initiated from the centre of the cell propagating outwards. Z stack collected over 12 seconds [Max projected 32 µm] and played at 120 times normal speed.

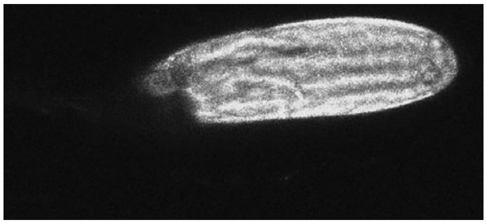

Movie 3. A mature oocyte expressing UAS-*myrGCaMP5* following the addition of activation buffer. Posterior initiation of the Ca^{2+} wave with secondary propagation from the anterior and lateral cortex. Recovery is initiated from both poles. Z stack collected over 20 seconds [Max projected 38 µm] and played at 200 times normal speed.

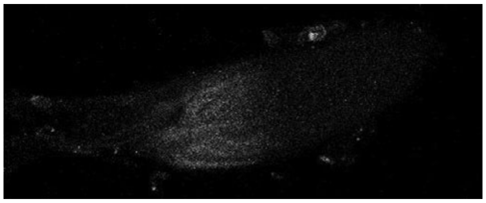

Movie 4. A mature oocyte expressing UAS-*myrGCaMP5* cultured in activation buffer with 10 µg/ml cytochalasin-D. Intracellular Ca^{2+} increases from the posterior pole as in wild-type but fails to propagate across the entire cell. A similarly compromised anterior wave also fails to propagate fully. Z stack collected over 30 seconds [Max projected 41.5 µm] and played at 300 times normal speed (corresponding to Fig. 6C).
